# *Ageratina adenophora* (Spreng.) King & H. Rob. Standardized leaf extract as an antidiabetic agent for type 2 diabetes: An *in vitro* and *in vivo* evaluation

**DOI:** 10.3389/fphar.2023.1178904

**Published:** 2023-04-17

**Authors:** Khaidem Devika Chanu, Nanaocha Sharma, Vimi Kshetrimayum, Sushil Kumar Chaudhary, Suparna Ghosh, Pallab Kanti Haldar, Pulok K. Mukherjee

**Affiliations:** ^1^ Institute of Bio-resources and Sustainable Development (IBSD), Imphal, Manipur, India; ^2^ School of Biotechnology, Kalinga Institute of Industrial Technology (KIIT), Deemed to Be University, Bhubaneswar, Odisha, India; ^3^ School of Natural Product Studies, Department of Pharmaceutical Technology, Jadavpur University (JU), Kolkata, West Bengal, India

**Keywords:** antidiabetic activity, *Ageratina adenophora*, streptozotocin–nicotinamide, type 2 diabetes, chlorogenic acid, caffeic acid

## Abstract

Type 2 diabetes has become one of the major health concerns of the 21st century, marked by hyperglycemia or glycosuria, and is associated with the development of several secondary health complications. Due to the fact that chemically synthesized drugs lead to several inevitable side effects, new antidiabetic medications from plants have gained substantial attention. Thus, the current study aims to evaluate the antidiabetic capacity of the *Ageratina adenophora* hydroalcoholic (AAHY) extract in streptozotocin–nicotinamide (STZ–NA)-induced diabetic Wistar albino rats. The rats were segregated randomly into five groups with six rats each. Group I was normal control, and the other four groups were STZ–NA-induced. Group II was designated diabetic control, and group III, IV, and V received metformin (150 mg/kg b.w.) and AAHY extract (200 and 400 mg/kg b.w.) for 28 days. Fasting blood glucose, serum biochemicals, liver and kidney antioxidant parameters, and pancreatic histopathology were observed after the experimental design. The study concludes that the AAHY extract has a significant blood glucose lowering capacity on normoglycemic (87.01 ± 0.54 to 57.21 ± 0.31), diabetic (324 ± 2.94 to 93 ± 2.04), and oral glucose-loaded (117.75 ± 3.35 to 92.75 ± 2.09) Wistar albino rats. The *in vitro* studies show that the AAHY extract has α-glucosidase and α-amylase inhibitory activities which can restore the altered blood glucose level, glycated hemoglobin, body weight, and serum enzymes such as serum glutamic pyruvic transaminase, serum glutamic oxaloacetic transaminase, serum alkaline phosphatase, total protein, urea, and creatinine levels close to the normal range in the treated STZ–NA-induced diabetic rats. The evaluation of these serum biochemicals is crucial for monitoring the diabetic condition. The AAHY extract has significantly enhanced tissue antioxidant parameters, such as superoxide dismutase, glutathione, and lipid peroxidation, close to normal levels. The presence of high-quantity chlorogenic (6.47% w/w) and caffeic (3.28% w/w) acids as some of the major phytoconstituents may contribute to the improvement of insulin resistance and oxidative stress. The study provides scientific support for the utilization of *A. adenophora* to treat type 2 diabetes in the STZ–NA-induced diabetic rat model. Although the preventive role of the AAHY extract in treating Wistar albino rat models against type 2 diabetes mellitus is undeniable, further elaborative research is required for efficacy and safety assessment in human beings.

## 1 Introduction

A significant global health concern of the 21st century is the incidence of type 2 diabetes mellitus (T2DM) or diabetes mellitus (DM), which has reached an epidemic level (Chan et al., 2005). Hyperglycemia, hyperlipidemia or dyslipidemia, and glycosuria are the hallmarks of diabetes mellitus. It is a complex and multifactorial metabolic condition typically brought on by faulty protein, carbohydrate and fat metabolism, damaged pancreatic beta cells, insulin resistance, or insulin insufficiency (Oguntibeju, 2019). This metabolic condition led to increased blood glucose levels (BGLs) and develops into chronic, life-threatening microvascular, macrovascular, and neuropathic consequences over time. Nephropathy, retinopathy, cataract, neuropathy, cardiovascular, stroke, coronary artery diseases, and food-related diseases are among the many consequences linked to DM (Khursheed et al., 2019; Padhi et al., 2020). In 2017, it was projected that 415 million people have diabetes globally. By 2025, there will be 300 million adult cases, which are still expected to upsurge till 693 million by 2045. According to the predicted diabetic population, more than half (49.7%) are undiagnosed (Liu et al., 2013; Cho et al., 2018). In surge of the increasing incidence of DM, it becomes a necessity to curb this metabolic disorder. Several marketed antidiabetic drugs are in use with a goal to suppress the disease progression on a global scale. However, due to affordability and safety concerns, many researchers are inclined toward naturally occurring antidiabetic bioactive compounds from plant sources.

Biological-derived or chemical drugs such as sulfonylureas, thiazolidinediones, biguanides, meglitinides, α-glucosidase inhibitors, dipeptidyl peptidase-4 (DPP-4) inhibitors, glucagon-like peptide-1 (GLP1) receptor agonists, and dopamine D2-receptor agonists are currently the principal antidiabetic medications for diabetes mellitus (Blonde, 2009; Tahrani et al., 2011; He et al., 2019). Sulfonylureas thiazolidinediones, biguanides, and meglitinides act by stimulating insulin production; α-glucosidase inhibitors halt carbohydrate breakdown, thereby enhancing glycemic index; DPP-4 inhibitors and GLP1 receptor agonists enhance insulin production, while inhibiting glucagon secretion from pancreatic islets of Langerhans; and dopamine D2-receptor agonists enhance the glycemic index via activation of hypothalamic dopamine D2-receptors (Dahlén et al., 2022). The recovery of diabetic patients is, however, significantly hampered by several unfavorable side effects and poor efficacy of these hypoglycemic medications. For instance, trodusquemine, which is a protein tyrosine phosphatase 1B (PTP-1B) inhibitor drug, has been disapproved by the Food and Drug Administration (FDA) for lower selectivity and adverse effects. Sodium-glucose co-transporter 2 (SGLT2) inhibitor drugs such as dapagliflozin, canagliflozin, and empagliflozin possess several side effects such as dehydration, diabetic ketoacidosis, vaginal yeast infections, urinary tract infections, joint ache, Fournier’s gangrene, and low blood pressure (Dowarah and Singh, 2020). Sulfonylureas, thiazolidinediones, α-glucosidase inhibitors, and biguanides can lead to hypoglycemic risk, weight gain, hepatotoxicity, gastrointestinal disorders, and lactic acidosis (Feldman, 1985; Lebovitz and Banerji, 2001; Carles et al., 2008; Chaudhury et al., 2017). DPP-4 inhibitors can also result in nausea, nasopharyngitis, headache, and hypersensitivity. Despite the fact that incretin-based medications have many advantages, they are nonetheless accompanied by significant gastrointestinal issues such as nausea, sour stomach, indigestion, belching, vomiting, and diarrhea (Drucker, 2006). There are several synthesized FDA-approved drugs which have made substantial improvement with time and has helped millions of T2DM patients as well as manage secondary complications. However, the dynamic nature of drug approval and withdrawal occurs as many drugs are more or less accompanied by certain deleterious effect in the long run (Dahlén et al., 2022). Therefore, the development of safer and more effective treatment medications is still critically needed.

Fortunately, many naturally occurring antidiabetic bioactive agents have never lost their effectiveness and continue to be a key component in the treatment as well as anti-T2DM drug discovery (Xu et al., 2018). Due to their affordability and safety concerns, almost 80% of people use plant-based traditional medicines (Ekor, 2014). A survey found that over a thousand plant species are utilized as a traditional folk treatment for diabetic mellitus (Osadebe et al., 2014). The primary medication on the market for the management of type 2 diabetes, metformin, is likewise made from guanidines that are extracted from the *Galegine officinalis* plant. For over 60 years, several new therapeutic antidiabetic drugs have been introduced; however, metformin is prioritized for T2DM patients, considering its safety profile and also affordability compared to other newer alternatives such as SGLT2 inhibitors as well as GLP1 receptor agonists (Bailey and Day, 2004; Triggle et al., 2022).

*Ageratina adenophora* (Spreng.) R. King & H. Robinson, also known as *Eupatorium adenophorum* Spreng., originated in Mexico and Costa Rica, belongs to the Asteraceae family and is widely distributed in Southeast Asian countries such as India, Pakistan, China, Nepal, Singapore, Thailand, Malaysia and the Philippines, New Zealand, eastern Australia, Northern America, and South Africa (Wan et al., 2010). The plant is commonly referred to as Crofton weed, eupatory, sticky snakeroot, Mexican devil, and Banmara (Muniappan et al., 2009), and in Manipur, India, the plant is locally called Naga mana or Japanpu (Ringmichon and Gopalkrishnan, 2017). It has been observed that the plant is an herbaceous perennial invasive weed and possesses several secondary metabolites that are pharmacologically intriguing, including terpenoids, alkaloids, polyphenols, saponins, flavonoids, coumarins, phenylpropanoids, steroids, and phenolic acids (Liu et al., 2015). Several pharmacological studies showed that *A. adenophora* extract has various biological therapeutic properties such as antiviral, antiinflammatory, wound-healing, antioxidant, antibacterial, antipyretic, wound-healing, and analgesic properties (André et al., 2019; Poudel et al., 2020). The leaves and tender parts of the plant have abundant chlorogenic acid or 5-O-caffeoylquinic acid (C16 H18 O9, 354.31 g/mol) (Liu et al., 2016). The chlorogenic acid is an ester of caffeic acid (C9 H8 O4, 180.16 g/mol) and quinic acid, which has attracted substantial attention due to its antioxidant, antiinflammatory, antimicrobial, antilipidemic, antihypertensive, and antitumor properties (Santana-Gálvez et al., 2017; Naveed et al., 2018). There are several antidiabetic reports of chlorogenic (Cho et al., 2010; Meng et al., 2013) and caffeic acids (Zhao et al., 2022) used as novel insulin sensitizers; they improve glucose tolerance, insulin resistance, and cellular oxidative stress and manage obesity. It has been reported that caffeic acid shows prophylactic activity against diabetic kidney disease by suppressing autophagy regulatory miRNAs (miR-133b, miR-342, and miR-30a) in high-fat diet streptozotocin-induced diabetic rats (Matboli et al., 2017). Caffeic acid and a majority of its derivatives reduce oxidative stress, manage hyperglycemic condition, and also aid in improving secondary complications associated with DM (Ganguly et al., 2023).

The main aim of the current study is to assess the antidiabetic capacity of *A. adenophora* hydroalcoholic extract in STZ–NA-induced diabetic rats. Ageratina of different species, *Ageratina grandifolia* and *Ageratina petiolaris*, have been cited to possess α-glucosidase inhibitory potential (Gutiérrez-González et al., 2021) and induces hypoglycemic effect (Bustos-brito et al., 2016; Mata-torres and Andrade-cetto, 2020). A report has mentioned the traditional use of *A. adenophora* leaves to treat diabetes in Nigeria (Awah et al., 2012); however, further scientific validation has not been carried out. Another recent work on the *in vitro* antidiabetic activity of *A. adenophora* methanolic extract from Nepal reported an α-amylase inhibitory activity, but further assessment *in vivo* was not implemented (Kapali and Sharma, 2021). Although few preliminary studies have mentioned the antidiabetic effect of *A. adenophora* extract, further extensive validation with animal models is crucial for the development of new drugs. The plant is extensively grown in Manipur as an unattended herb. In spite of the abundance, its therapeutic potential has not been utilized adequately due to lack of limited knowledge. Therefore, considering the preliminary works and reported scientific significances of *A. adenophora*, the current study has been performed to evaluate the phytochemicals, antioxidant, and *in vitro* as well as *in vivo* antidiabetic properties of the plant.

## 2 Materials and methods

### 2.1 Materials

α-Glucosidase (from *Saccharomyces cerevisiae*), α-amylase (from *Bacillus subtilis*) and p-nitrophenyl-α-D-glucopyranoside (pNPG), streptozotocin (STZ), chlorogenic acid (≥95%), and caffeic acid (≥98.0%) were procured from Sigma-Aldrich Co. (St. Louis, United States). Nicotinamide (NA) and metformin hydrochloride were from Hi media. All other reagents and chemicals used for the study were of analytical grade.

### 2.2 Plant materials and extraction

The fresh leaves and aerial parts of the plants were collected from Mao, Manipur (latitude: 25°30′24.69″N and longitude: 94°08′03.01″E), during the month of January 2020, growing at an altitude of 1665 m above the sea level. The taxonomic identification of the herbarium was authenticated by the Institute of Bioresources and Sustainable Development (IBSD), India, such as *A. adenophora* (Spreng.) R. M. King & H. Rob., and deposited at the Plant Systematic and Conservation Laboratory, IBSD (Herbarium No. Institute of Bioresources and Sustainable Development/M-274)**.** The collected sample was washed, shade-dried, powdered, and kept for a week by macerating with methanol:water (70:30 v/v), and the macerate was filtered at the end of the time point. Finally, the filtrate was evaporated with the help of a rotary vacuum evaporator (IKA RV 10) set at 45°C, followed by lyophilization (Scanvac cool safe, labogene scandinavian by design, Denmark) (Alamgeer et al., 2013). In total, 132.3 gm dried crude extract was yielded from 1,500 gm of dried leaf powder. The % yield of the *A. adenophora* hydroalcoholic (AAHY) extract was calculated as

$$Yield\left(\%\right)=\frac{X}{Y}\times 100,$$ (1)

where X is weight of the dried crude extract obtained and Y is weight of dried leaves powder used for extraction.

### 2.3 Qualitative phytochemical analysis

To examine the presence or absence of the major phytochemical group of compounds in the AAHY extract, such as alkaloids, phenols, flavonoids, saponins, tannins, glycosides, terpenoids, quinones, and steroids, we followed standard protocols for screening preliminary qualitative phytochemical profiling (Banu and Cathrine, 2015; Sisay et al., 2022).

### 2.4 High-performance thin-layer chromatography analysis of the AAHY extract

The percentage content of standard chlorogenic acid and caffeic acid in the AAHY extract was estimated by the high-performance thin-layer chromatography (HPTLC) comparative analysis method with the respective retardation factor (*Rf*) of the standard phytoconstituents. The Camag HPTLC instrument (Muttenz, Switzerland) was used for the analysis of samples. The standard stock solution (1 mg/mL) of chlorogenic acid and caffeic acid was prepared by dissolving 1 mg accurately weighed standard in 1 mL HPLC-grade methanol. All the solutions were vortexed and kept in an ultrasonic bath till dissolved and filtered through a 0.45 μ syringe filter before analysis. The external standard calibration curve for chlorogenic acid and caffeic acid was prepared in a concentration range from 20 to 100 μg/mL and 100 to 180 μg/mL, respectively. Then, the solutions were drawn into a CAMAG LINOMAT V applicator fitted out with a syringe and spotted on aluminum-backed HPTLC plates 10 × 10 cm with 0.2 mm layers of silica gel 60 F_254_. Then, the plates were developed using a suitable mobile phase. The detection of the compounds was performed at 302 nm. The amount of chlorogenic acid and caffeic acid present in the sample was determined through the construction of a calibration curve by plotting the peak area against corresponding concentrations by means of linear regression using visionCATS 3.0 software (Orfali et al., 2021; Chaudhary et al., 2023).

### 2.5 *In vitro* antioxidant capacity of the AAHY leaf extract

#### 2.5.1 DPPH radical scavenging activity

Measurement of the scavenging effect on 2,2-diphenyl-1-picrylhydrazyl (DPPH) (Sigma-Aldrich) was performed according to the work of Amorati and Valgimigli (2018). 100 μl of 0.2 mM DPPH was prepared in methanol and was mixed with 100 μL of different concentrations of the AAHY extract (1.0, 2.5, 5.0, 10.0, 15.0, 30.0, 60.0, and 100.0 μg/mL). The reaction mixture was shaken well and incubated for 30 min in the dark, and then, the absorbance was measured at 517 nm using a Varioskan LUX multimode microplate reader (ESW version 1.00.38) from Thermo Fisher Scientific.

The percentage of DPPH free radical scavenging capacity was calculated using the following equation:

$$DPPH\;free\;radical\;scavenging\;capacity\left(\%\right)=\frac{\left(Acontrol-Asample\right)}{Acontrol}\times 100,$$ (2)

where Acontrol is the absorbance of DPPH mixed with methanol and Asample is the absorbance of DPPH mixed with the sample AAHY extract. Experiments were performed thrice (*n* = 3). L-ascorbic acid (Sigma-Aldrich) was used as positive control.

#### 2.5.2 ABTS cation radical scavenging activity

The ABTS radical cation scavenging capacity of the AAHY extract was assayed with 2,2’-azinobis-3-ethylbenzothiazoline-6-sulfonic acid (ABTS) (Sigma-Aldrich) following the work of Floegel et al. (2011). The ABTS radical cation solution was prepared by mixing 7.4 mmol/L ABTS and 2.6 mmol/L potassium persulfate (Sigma-Aldrich). 100 μL of the prepared ABTS radical cation solution was mixed with 100 μL of different concentrations of the AAHY extract (1.0, 2.5, 5.0, 10.0, 15.0, 30.0, 60.0, and 100.0 μg/mL), and after 6 min incubation time, the absorbance was measured at 734 nm using a Varioskan LUX multimode microplate reader (ESW version 1.00.38) from Thermo Fisher Scientific.

The percentage of ABTS^+^ free radical scavenging capacity was calculated using the following equation:

$$ABTS^{+}radical\;scavenging\;capacity\left(\%\right)=\frac{\left(Acontrol-Asample\right)}{Acontrol}\times 100,$$ (3)

where Acontrol is the absorbance of the ABTS with methanol and Asample is the absorbance of the ABTS mixed with the sample AAHY extract. Experiments were performed thrice (*n* = 3). L-ascorbic acid was used as positive control.

### 2.6 *In vitro* antidiabetic activity

#### 2.6.1 α-Glucosidase inhibition assay

The α-glucosidase inhibition assay was performed following methods previously described by Yao et al. (2013); Kim et al., 2004. Different concentrations of standard inhibitor acarbose and the sample AAHY extract (20, 40, 60, 80, 100, and 120 μg/mL) were prepared. Then, 50 µL of 0.1 M potassium phosphate buffer (pH: 6.8) and 10 µL of alpha-glucosidase (1 U/mL) were mixed and incubated. After 20 min of incubation at 37°C, 20 µL of p-nitro phenyl glucopyranoside (pNPG, 5 mM) was added, mixed well, and re-incubated at 37°C for 30 min. The reaction was stopped by adding 40 µL of 0.1 M Na2 CO3 solution. The enzyme activity was estimated by measuring the absorbance of the end product p-nitrophenol at 410 nm using a microplate reader (Varioskan LUX multimode microplate reader, ESW version 1.00.38) from Thermo Fisher Scientific. The inhibition assay was performed thrice, and the percentage of inhibition was calculated as follows:

$$\alpha -Glucosidase\;Inhibitory\left(\%\right)=\left(1-\frac{Asample}{Acontrol}\right)\times 100,$$ (4)

where Asample is the absorbance in the presence of both α-glucosidase and sample and Acontrol is the absorbance of the reaction mixture containing the same volume of buffer solution instead of the sample.

#### 2.6.2 α-Amylase inhibition assay

The α-amylase inhibition assay was carried out following the work of Yao et al. (2013) and Telagari and Hullatti (2015). Different concentrations (20, 40, 60, 80, 100, and 120 μg/mL) of standard acarbose and the sample AAHY extract were prepared. 50 μL of sodium phosphate buffer (100 mM, pH 6.8) was mixed with 10 µL α-amylase (2U/mL) soluble starch (1%). After 30 min of incubation at 37°C, 20 µL substrate, 1% soluble starch prepared in phosphate buffer 100 mM (pH: 6.8) was added and further re-incubated at 37°C for 30 min. The reaction was terminated by adding 100 µL dinitrosalicylic acid reagent solution and boiling for 10 min. The enzyme activity was estimated by measuring the absorbance at 540 nm using a microplate reader (Varioskan LUX multimode microplate reader, ESW version 1.00.38) from Thermo Fisher Scientific. The inhibition assay was performed thrice, and the inhibition percentage was calculated as follows:

$$\alpha -Amylase\;Inhibitory\left(\%\right)=\left(1-\frac{Asample}{Acontrol}\right)\times 100,$$ (5)

where Asample is the absorbance in the presence of both α-glucosidase and sample and Acontrol is the absorbance of the reaction mixture containing the same volume of buffer solution instead of the sample.

### 2.7 *In vivo* antidiabetic activity

#### 2.7.1 Experimental animals

Healthy normoglycemic (80–90 mg/dL) adult male Wistar albino rats (150–200 g) obtained from the registered breeder–Saha Enterprise, Kolkata (Reg. No. 1828/PO/BT/S/15/CPCSEA), were used for the experimental study. Rats were maintained in polypropylene cages bedded with straws under standard ambient conditions (temperature 25°C ± 4°C with 12/12 h light/dark cycle; 50—70 humidity). The rats were fed on a standard pellet diet and given free access to water *ad libitum*. All the experimental protocols were scrutinized and approved by the university’s animal ethical committee (Ref. No. ACE/PHARM/1502/09/2015, Jadavpur University, Kolkata 700032, West Bengal, India).

#### 2.7.2 Acute toxicity study

Swiss albino mice were used to evaluate the acute oral toxicity test of the AAHY extract following instructions by the Organization of Economic Cooperation and Development (OECD), Guideline 425. The animals were observed for general behaviors such as tremors, aggressiveness, hypnosis, convulsions, diarrhea, analgesia, and skin color for the first 24 h after administration of the test sample AAHY extract at a limit dose of 2000 mg/kg b.w (OECD, 2022).

#### 2.7.3 Diabetes induction

Induction of diabetes by intraperitoneal (i.p) injection of streptozotocin (STZ) was given to the 16 h fasted rats; however, water was provided. STZ at a dose of 50 mg/kg b. w was dissolved in 0.1 Mcold citrate buffer (pH 4.5) just before administration, and an i. p injection of NA (100 mg/kg b. w) was given 15 min prior to STZ injection. Glucose solution 20% was provided for the first 24 h to STZ–NA-injected rats to prevent initial hypoglycemic mortality. The diabetic condition was confirmed by fasting blood glucose (FBG) measurement of blood drawn from the tail vein using a glucometer (ACCU-Chek active), and FBG ≥250 mg/dL was selected for the experiment (Ghasemi et al., 2014; Sisay et al., 2022).

#### 2.7.4 Extract effect on the blood glucose level of normoglycemic rats

Normal healthy rats (80–90 mg/dL) were fasted overnight for 16 h, but water was provided *ad libitum*. The normal control animals received normal saline water, and the treated groups were given predetermined doses of 200 and 400 mg/kg b.w., p.o., of the AAHY extract. The baseline blood glucose level of each rat was measured just prior to treatment (0 min) and after administration at 1, 2, 4, and 6 h, with the blood drawn from the tail vein under aseptic conditions (Birru et al., 2015; Sisay et al., 2022).

#### 2.7.5 Extract effect on blood glucose after the oral glucose tolerance test

Normal healthy rats (80–90 mg/dL) which were fasted for 16 h were used for the oral glucose tolerance test (OGTT). The rats were distributed into groups of three, and six rats were placed in each group (*n* = 6). Group I, designated as the normal control group, was given distilled water (5 mL/kg b.w., p.o.), and group II and III were treated with doses of 200 and 400 mg/kg b.w., p.o., respectively. After 30 min of administration, the rats received glucose (2 g/kg b.w., p.o.). Measurement of blood glucose level was carried out just before (0 min) and after 30, 60, and 120 min following oral glucose administration (Kifle et al., 2020). The blood was drawn from the tail vein and measured using a glucometer (ACCU-Chek active).

#### 2.7.6 Experimental design for antidiabetic activity

The rats were divided into five groups, with six rats in each group (*n* = 6). The experimental study was set up for 28 days.

Group I: Normal control rats received normal saline (0.5 mL/kg, b.w., p.o.).

Group II: The diabetic control group treated with STZ (50 mg/kg, b.w., i.p.) and NA (100 mg/kg, b.w., i.p.).

Group III: STZ–NA-induced diabetic rats treated with the AAHY extract (200 mg/kg b.w.), administered orally for 28 days.

Group IV: STZ–NA-induced diabetic rats treated with the AAHY extract (400 mg/kg b.w.) administered orally for 28 days.

Group V: STZ–NA-induced diabetic rats treated with metformin (150 mg/kg, p.o.) for 28 days.

#### 2.7.7 Glycated hemoglobin estimation

A commercially available glycated hemoglobin kit based on the ion exchange resin method [Coral clinical systems, a Division of Tulip Diagnostics (p) Ltd.] was used to measure glycated hemoglobin (HbA1c) levels in whole blood samples.

#### 2.7.8 Serum biochemical parameter determination

At the end of the experimental design, the rats were fasted overnight for 16 h and on the 29th day, they were anaesthetized using isoflurane and sacrificed by cervical dislocation. The blood sample was drawn and collected from the heart by cardiac puncture. Serum was acquired by centrifugation at 3,000 rpm for 10 min. An array of biochemical parameters such as serum glutamic pyruvic transaminase (SGPT), serum glutamic oxaloacetic transaminase (SGOT), serum alkaline phosphatase (SALP), total protein (TP), creatinine, and urea were measured with the collected serum using commercially available assay kits (Arkray Healthcare Pvt., Ltd., Surat, Gujarat, India).

#### 2.7.9 Serum lipid profile evaluation

Estimation of serum lipid profiles such as total cholesterol (TC), high-density lipoprotein (HDL), and triglycerides (TGs) were determined using commercially available kits (Arkray Healthcare Pvt., Ltd., Surat, Gujarat, India).

#### 2.7.10 Tissue antioxidant parameter estimation

The organs, liver and kidney, were carefully harvested from the rats and cleaned in ice-cold saline to remove blood. The organs were weighed, cut into pieces, and homogenized with phosphate buffer (0.025 M, pH 7.4). The homogenate was centrifuged for 15 min at 10,000 rpm at 4°C. The supernatant was collected and used for estimations of antioxidant parameters such as lipid peroxidation (LPO), reduced glutathione (GSH), and superoxide dismutase (SOD).

Lipid peroxidation (LPO) levels from the supernatant of liver and kidney tissues were determined as per the standard protocols followed by Ohkawa et al. (1979) and Niehaus and Samuelsson (1968). The supernatant, 0.02 M phosphate buffer saline (PBS), and 10% trichloro acetic acid (TCA), in the ratio of 1:1:2 were mixed and incubated for 30 min at room temperature. Then, the mixture was centrifuged at 3,000 rpm

For 10 min, 1mL of the supernatant was collected and mixed with 250 µL of 1% thiobarbituric acid (TBA) and heated for 60 min at 95°C until a stable pink color was observed. The OD was measured at 532 nm against an appropriate blank and expressed as µM of malondialdehyde (MDA)/mg protein.

The glutathione (GSH) levels of the liver and kidney tissues were assayed according to the methods of Ellman (1959) and Moron et al. (1979). 0.1 mL of respective homogenates mixed with 2.4 mL EDTA were incubated in ice for 10 min, followed by precipitation with 0.5 mL of 50% TCA. The precipitate was discarded by centrifugation at 4°C, 3,000 rpm for 15 min. To 1 mL of the clear supernatant, 1 mL of tris buffer and 0.05 mL of DTNB were added. Absorbance at 412 nm was measured after the reaction mixture had been incubated for 3 min. The amount of glutathione was expressed as µM of GSH utilized/mg protein.

The superoxide dismutase (SOD) activity levels of the tissue supernatants were assayed following the work of Marklund and Marklund (1974) and Kakkar et al. (1984). The reaction mixture contained 600 µL PBS, 60 µL of 186 µM phenazine methosulphate (PMS), 150 µL of 300 µM nitroblue tetrazolium (NBT), and 100 µL each of supernatant and NADH (780 µM). The reaction mixture was incubated for 90 s at 30°C, and the reaction was terminated by adding 500 µL of glacial acetic acid. The absorbance was measured at 560 nm against an appropriate blank and expressed as units/mg protein.

#### 2.7.11 Histopathological studies

The pancreas was carefully harvested from the euthanized rats after the termination of the experiment and subjected to histopathological studies. The tissues were washed with a standard saline solution for 5 min each, followed by 10% formalin fixation for 24 h, then dehydrated by passing through different alcohol solutions successively, and finally, embedded in paraffin. The embedded samples were sliced into ultra-thin (4–5 µm) sections with a semi-automated Thermo Scientific microtome. The sliced tissue samples were gently placed over warm water and carefully glided onto glass slides. The slides were deparaffinized, and the transparent intact tissue sections were stained with hematoxylin–eosin dye to provide structural contrast (Alturkistani et al., 2015). One or two drops of DPX mounting medium were spread over the tissue specimens on the slides and carefully covered with coverslips for preservation. The prepared stained slides were observed and photographed with the camera attached to the microscope (Nikon eclipse Ni–U). The size of the pancreatic islets from the captured histopathology image was calculated using ImageJ analysis software. The changes in the size have been expressed as relative % compared to the normal control group.

### 2.8 Statistical analysis

The results were evaluated using the statistical tool, one-way analysis of variance (ANOVA) *post hoc* Dunnett’s test using Graph pad prism 8.4.3 software (Graph Pad Software, San Diego United States). The size of the histopathology image was analyzed using ImageJ software. The data were represented as mean ± standard error of mean (SEM). The statistical significance was set at *p* < 0.05.

## 3 Results

### 3.1 Extraction yield of the AAHY extract

The yield of the AAHY extract was 8.82%.

### 3.2 Qualitative phytochemical analysis

The outcomes of the types of tests performed, the changes observed, and the inferences of the preliminary phytoconstituent analysis are summarized in Table 1.

**TABLE 1:** Preliminary qualitative phytochemical analysis of the AAHY extract.

Phytochemicals	Tests performed	Appearance	Inference
Alkaloids	Mayer’s	Reddish brown precipitate seen	+
Phenolics	Ferric chloride	Blue–black color seen	+
Flavonoids	Lead acetate	Yellow precipitate formed	+
Tannins	Braemer’s	Bluish-green color seen	+
Glycosides	Keller–Kiliani	Brown ring at the interface seen	+
Saponins	Foam test	Foam observed	−
Terpenoids	Salkowski’s	Dark bluish black observed	+
Quinones	Borntrager’s	Red color seen	+
Steroids	Liebermann–Burchard	Pink-to-reddish color not seen	−

(+): detected; (−): not detected.

### 3.3 HPTLC analysis of the AAHY extract

**FIGURE 1:**
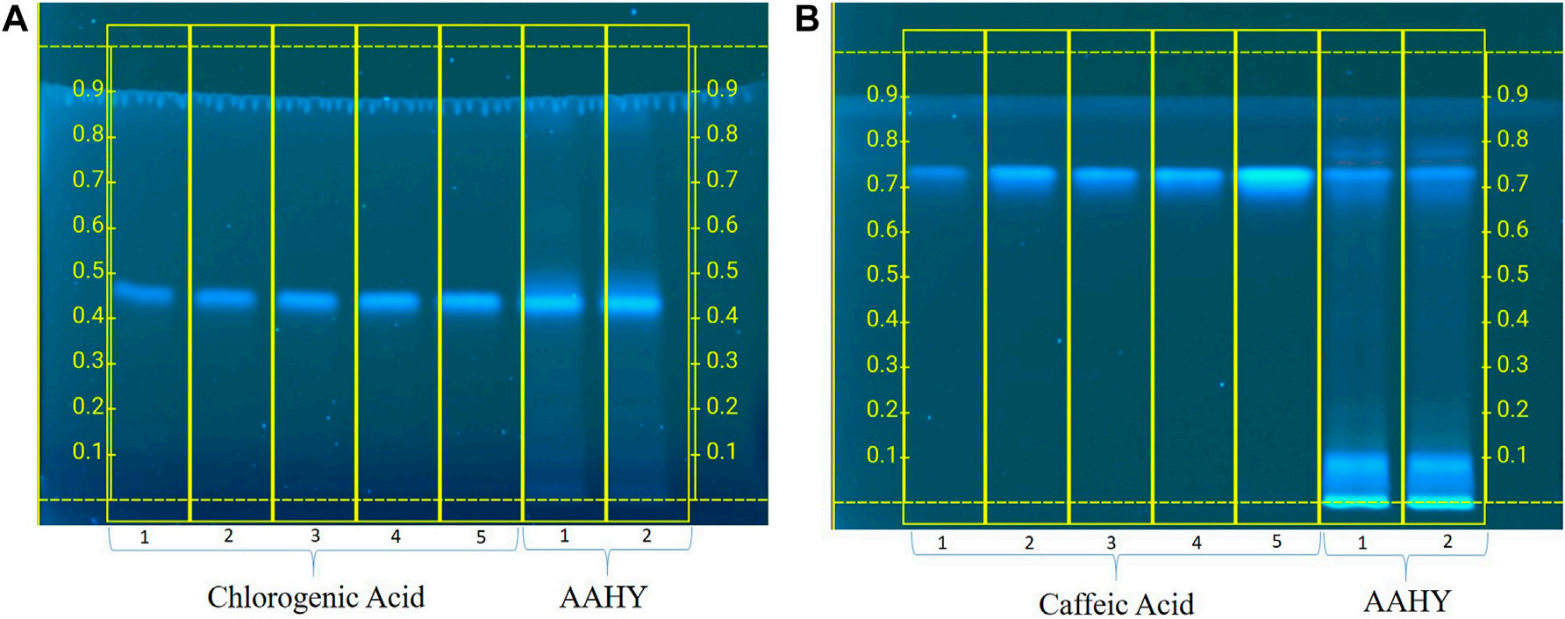
Standardization of the AAHY extract for the presence of **(A)** chlorogenic acid and **(B)** caffeic acid as the standard phytomarker through HPTLC analysis.

The developed plate was scanned 302 nm using a CAMAG TLC Scanner IV in the absorbance mode. The picture of the developed plate was captured by using CAMAG Reprostar 3. The photo documentation was carried out using the CAMAG TLC visualizer 2 under white, 254 and 366 nm (Figure 1). The standard compound (chlorogenic acid and caffeic acid) expressed a good linearity between concentrations and the peak area. The mobile phase toluene–ethyl acetate–formic acid (5:4:1, v/v) and ethyl acetate–acetic acid–formic acid–water (100:1.1:1.1:2.6, v/v) were found to produce a compact spot for chlorogenic acid and caffeic acid at *Rf* 0.43 and 0.72, respectively. A good linear precision relationship between the concentrations and peak areas was obtained with the correlation coefficient (*r* ^2^) > 0.997 and 0.998 for chlorogenic acid and caffeic acid, respectively. The amount of chlorogenic acid and caffeic acid was found to be 6.47% and 3.28 w/w in the AAHY extract, respectively.

### 3.4 Antioxidant activity

**FIGURE 2:**
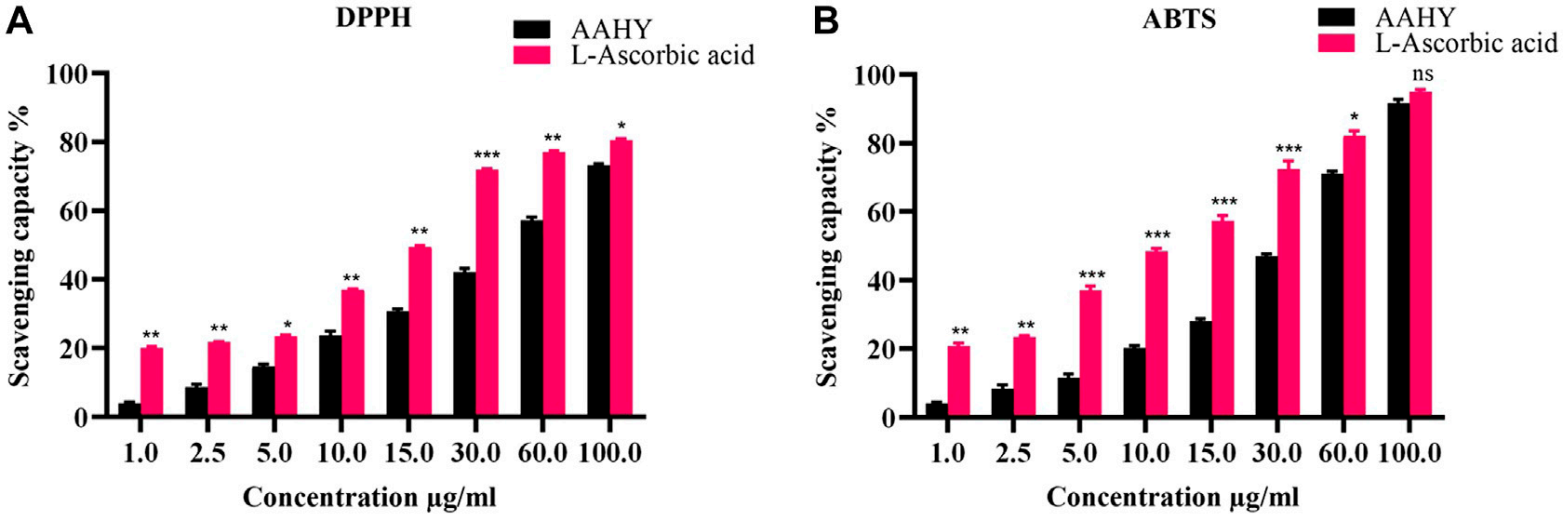
**(A)** DPPH and **(B)** ABTS free radical scavenging activities (%). A comparison of different concentrations of the AAHY extract and ascorbic acid is shown. Data are represented as mean ± SEM, and experiments were performed thrice (*n* = 3). *p < 0.05, **p < 0.01, and ***p < 0.001 versus the AAHY extract. SEM: standard error of mean.

The AAHY extract exerted DPPH as well as ABTS free radical scavenging activities in a dose-dependent manner Figure 1. The % inhibitory concentrations (IC_50_) of the AAHY extract and L-ascorbic acid were 44.52 ± 1.23 μg/mL and 15.12 ± 0.11 μg/mL, respectively, as determined by DPPH radical scavenging assay Figure 2A, whereas IC_50_ was 9.75 ± 0.33 μg/mL for L-ascorbic acid and 33.33 ± 0.66 μg/mL for the AAHY extract, respectively, when the scavenging capacity was assessed by ABTS cation radical scavenging assay Figure 2B.

### 3.5 *In vitro* α-glucosidase and α-amylase inhibition assays

**FIGURE 3:**
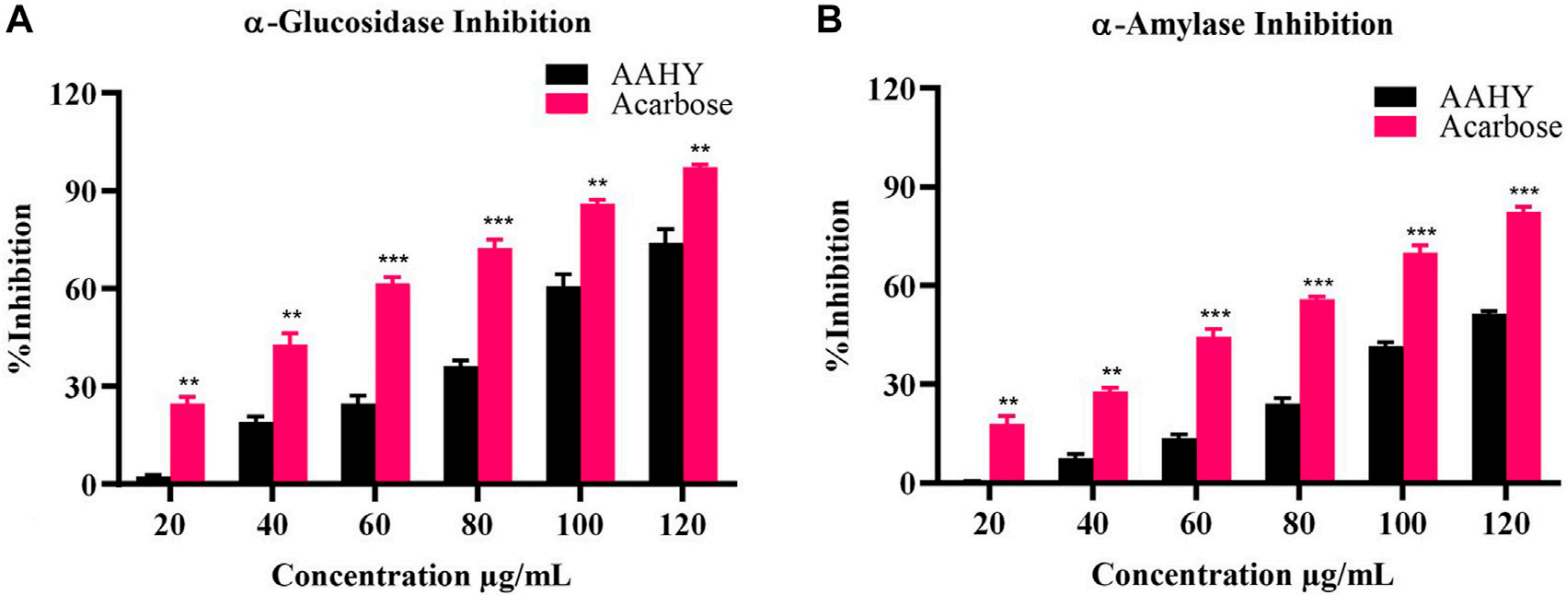
*In vitro* **(A)** α-glucosidase and **(B)** α-amylase activities of the AAHY extract. % inhibitions are expressed as mean ± SEM, and *n* = 3. *p < 0.05, **p < 0.01, and ***p < 0.001 versus the AAHY extract. SEM: standard error of mean.

The sample AAHY extract exhibited a dose-dependent inhibition of α-glucosidase and α-amylase activities. The inhibitory effects were compared with a standard inhibitor, acarbose. The α-glucosidase inhibition results of the extract and acarbose are shown in Figure 3A. The corresponding concentration for 50% inhibition (IC_50_) of the AAHY extract and acarbose was 93.47 ± 2.56 and 46.77 ± 1.31 μg/mL, respectively. α-Amylase inhibitory activities of the AAHY extract and acarbose are shown in Figure 3B, and the corresponding IC_50_ values were found to be 116.32 ± 1.15 and 70.62 ± 0.52 μg/mL, respectively.

### 3.6 Acute toxicity study

The study was performed by oral administration (p.o.) of the AAHY extract at a dose of 2000 mg/kg b.w. in mice. There were no toxicity signs, and none of the animals died till the end of the experiment. Since the animals were not toxic up to the dose of 2000 mg/kg b.w., further hypoglycemic experiments were carried out at doses of 200 and 400 mg/kg b.w, respectively.

### 3.7 Oral glucose tolerance test

The OGTT showed that the BGLs of normoglycemic rats were elevated in the first 30 min after the oral administration of glucose and gradually decreased. However, the AAHY extract (200 and 400 mg/kg b.w.) significantly reduced blood glucose levels at 60 and 120 min in glucose-loaded rats compared to untreated normal control rats (Table 2).

**TABLE 2:** Effect of the AAHY extract in the oral glucose tolerance test.

Groups	Fasting blood glucose level (mg/dL)			
	0 min	30 min	60 min	120 min
Normal control	84.33 ± 1.14	130.25 ± 3.09	123.5 ± 2.5	117.75 ± 3.35
AAHY extract 200 mg/kg	81.33 ± 2.81^ans^	126.75 ± 2.49^ans^	113.25 ± 2.39^a*^	104.5 ± 2.32^a**^
AAHY extract 400 mg/kg	82.16 ± 2.44^ans^	120.75 ± 2.52^a*^	107.75 ± 1.6^a**^	92.75 ± 2.09^a**^
Metformin 150 mg/kg	84 ± 1.36^ans^	108.25 ± 2.78^a**^	93.75 ± 2.86^a***^	86.5 ± 2.06^a***^

The values are represented as mean ± SEM, and *n* = 6 for each group.

^a^All treated groups versus the normal control group at the corresponding time point.

^ns^No significant difference was observed.

^ans^No significant difference observed when all treated groups were compared to the normal control group at the corresponding time point. SEM, standard error of mean; AAHY extract, *Ageratina adenophora* hydroalcoholic extract.*p < 0.05, **p < 0.01, and ***p < 0.001.

### 3.8 Effect of the AAHY extract on fasting blood glucose and body weight

The fasting blood glucose levels of STZ–NA-induced diabetic rats (group II) were significantly elevated compared to normal control rats (group I) during the experimental study. Daily oral administration of the AAHY extract at the doses of 200 mg/kg b.w. (group III) and 400 mg/kg b.w. (group IV) to the diabetic rats significantly reduced FBG near to the normal level (group I) compared to the diabetic control rats (group II) in a dose-dependent manner. The standard metformin (150 mg/kg b.w.)-treated rats (group V) showed reduced FBG compared to the diabetic control (Table 3).

**TABLE 3:** Effect of the AAHY extract on fasting blood glucose (mg/dL).

Groups	Fasting blood glucose level (mg/dL)				
	Day 0	Day 7	Day 14	Day 21	Day 28
I Normal control	93 ± 2.04	83 ± 1.04	89 ± 2.04	92 ± 2.21	96 ± 1.21
II Diabetic control	372 ± 2.18^a***^	346 ± 3.51^a***^	364 ± 2.74^a***^	349 ± 3.45^a***^	324 ± 2.94^a***^
III Diabetic + AAHY extract 200 mg/kg	354 ± 1.36^bns^	274 ± 2.89^b*^	155 ± 3.45^b**^	135 ± 1.01^b**^	103 ± 3.2^b***^
IV Diabetic + AAHY extract 400 mg/kg	337 ± 3.3^bns^	231 ± 1.5^b*^	120 ± 3.71^b**^	108 ± 2.48^b***^	93 ± 2.04^b***^
V Diabetic + metformin 150 mg/kg	349 ± 2.12^bns^	217 ± 3.47^b*^	122 ± 1.78^b**^	101 ± 2.69^b***^	84 ± 1.27^b***^

The data are represented as mean ± SEM, and *n* = 6 for each group.

^a^The normal control group versus the diabetic control group.

^b^All treated groups versus the diabetic control group at the corresponding time point.

^ns^No significant difference was observed.

^bns^No significant difference observed when all treated groups were compared to the diabetic control group at the corresponding time point. SEM, standard error of mean; AAHY extract, *Ageratina adenophora* hydroalcoholic extract; diabetic: streptozotocin (50 mg/kg, b.w.) + nicotinamide (100 mg/kg, b.w.). *p < 0.05, **p < 0.01, and ***p < 0.001.

The body weight of STZ–NA-induced diabetic rats (group II) gradually decreased, whereas the groups (III and IV) administered with the AAHY extract at the doses of 200 and 400 mg/kg b.w. showed gradual improvement as compared to normal control rats (group I). The rats treated with drug standard metformin at 150 mg/kg b.w. (group V) also exhibited a gradual increase in body weight. The study data are shown in Table 4.

**TABLE 4:** Effect of the AAHY extract on the body weight of experimental rats (g).

Groups	Body weight (g)				
	Day 0	Day 7	Day 14	Day 21	Day 28
I Normal control	179 ± 1.91	177 ± 1.96	173 ± 2.16	178 ± 1.28	181 ± 2.9
II Diabetic control	188 ± 2.19^ans^	169 ± 1.37^ans^	149 ± 3.08^a**^	132 ± 1.51^a**^	119 ± 2.98^a***^
III Diabetic + AAHY extract (200 mg/kg)	182 ± 2.08^bns^	167 ± 1.7^bns^	152 ± 0.93^bns^	149 ± 0.85 ^b*^	146 ± 3.02^b**^
IV Diabetic + AAHY extract (400 mg/kg)	185 ± 3.02^bns^	173 ± 1.51^bns^	159 ± 2.3^bns^	158 ± 0.97^b*^	162 ± 1.28^b**^
V Diabetic + metformin (150 mg/kg)	177 ± 1.97^bns^	169 ± 0.98^bns^	166 ± 1.87^b*^	164 ± 2.31^b**^	168 ± 1.97^b**^

The values are represented as mean ± SEM, and *n* = 6 for each group.

^a^The normal control group versus the diabetic control group.

^b^All treated groups versus the diabetic control group on the corresponding day.

^ns^No significant difference was observed.

^ans^No significant difference observed when all treated groups were compared to the normal control group at the corresponding time point.

^bns^No significant difference observed when all treated groups were compared to the diabetic control group at the corresponding time point. SEM, standard error of mean; AAHY extract, *Ageratina adenophora* hydroalcoholic extract; diabetic: streptozotocin (50 mg/kg, b. w.) + nicotinamide (100 mg/kg, b.w.). *p < 0.05, **p < 0.01, and ***p < 0.001.

### 3.9 Hypoglycemic activity on normoglycemic rats

There was no significant difference in blood glucose levels between the groups prior to treatments. In fact, 2 h after the administration of the extracts and metformin, the FBG level was lowered. Following 6 h of treatment, both the extracts (200 and 400 mg/kg) and metformin (150 mg/kg) were able to significantly deduce FBG levels of the normoglycemic rats compared to the 2 h time point (Table 5).

**TABLE 5:** Hypoglycemic activity of the AAHY extract in normoglycemic rats.

Group	Blood glucose level (mg/dL)				
	0 h	1 h	2 h	4 h	6 h
Normal control	86 ± 1.26	85.91 ± 0.4	87.58 ± 0.64	86.71 ± 1.21	87.01 ± 0.54
AAHY extract 200 mg/kg	87.41 ± 2.17^ans^	83.02 ± 1.05^ans^	78.2 ± 0.51^ans^	72.61 ± 0.28^a*^	69.48 ± 0.14^a*^
AAHY extract 400 mg/kg	82.9 ± 0.97^ans^	79.73 ± 0.21^ans^	67.54 ± 1.24^a*^	61.37 ± 0.57^a**^	57.21 ± 00.31^a**^
Metformin (150 mg/kg)	84.34 ± 0.82^ans^	75.41 ± 0.54^ans^	65.17 ± 1.27^a*^	57.24 ± 0.48^a**^	51.08 ± 0.16^a**^

The values are represented as mean ± SEM, and n = 6 for each group.

^a^All treated groups versus the normal control group at the corresponding time period.

^ns^No significant difference.

^ans^No significant difference observed when all treated groups were compared to the normal control group at the corresponding time point. SEM, standard error of mean; AAHY extract, *Ageratina adenophora* hydroalcoholic extract.*p < 0.05; **p < 0.01.

### 3.10 Glycated hemoglobin

**FIGURE 4:**
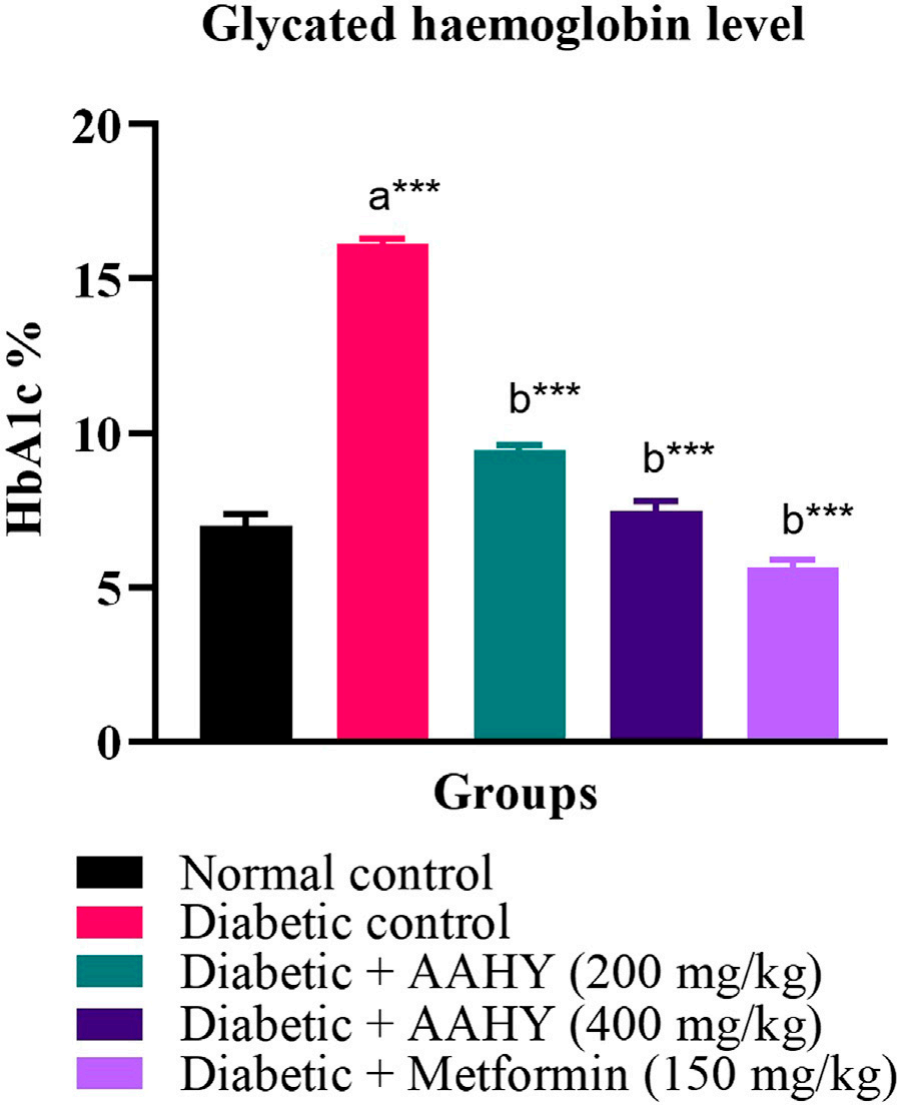
Effect of the AAHY extract on the glycated hemoglobin level. The error bar represents mean ± SEM, and *n* = 6 for each group. a represents the diabetic control group versus the normal control group, and b represents the diabetic control group versus treated groups. *p < 0.05, **p < 0.01, and ***p < 0.001. SEM: standard error of mean.

After the termination of the experiment, the blood samples were examined for glycated hemoglobin **(**HbA1c). The HbA1c level of the STZ–NA-induced diabetic control rats was elevated compared to normal control rats. However, the HbA1c level of the STZ–NA-induced diabetic rats treated with doses of the AAHY extract 200 and 400 mg/kg b.w and metformin 150 mg/kg b.w. was reduced compared to the diabetic control rats. The activity observed with the AAHY extract dose of 400 mg/kg b.w was comparable with the standard drug metformin (Figure 4).

### 3.11 Serum biochemical parameters

**FIGURE 5:**
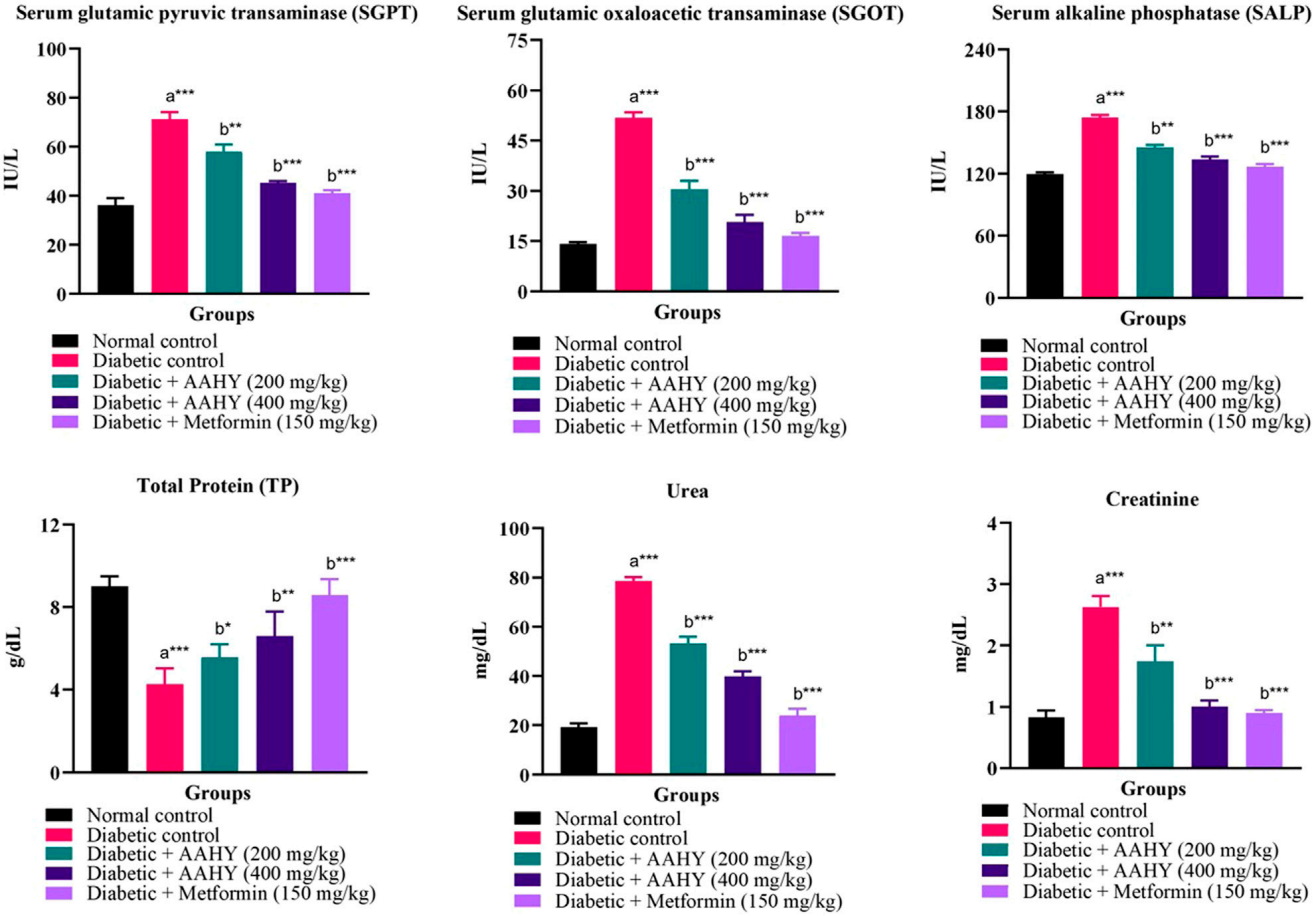
Effect of the AAHY extract on serum biochemical parameters. The error bar denotes mean ± SEM, and *n* = 6 for each group. a represents the diabetic control group versus the normal control group, and b represents the diabetic control group versus treated groups. *p < 0.05, **p < 0.01, and ***p < 0.001. SEM: standard error of mean.

The efficacy of the AAHY extract on serum biochemical parameters such as SGPT, SGOT, SALP, TP, creatinine, and urea is shown in Figure 3. In STZ–NA-induced diabetic rats, oral administration of the AAHY extract and metformin significantly reduced SGPT, SGOT, SALP, creatinine, and urea, when compared to diabetic control rats. However, the total protein (TP) content increased in the AAHY extract- and metformin-administered groups (Figure 5).

### 3.12 Serum lipid profile

**FIGURE 6:**
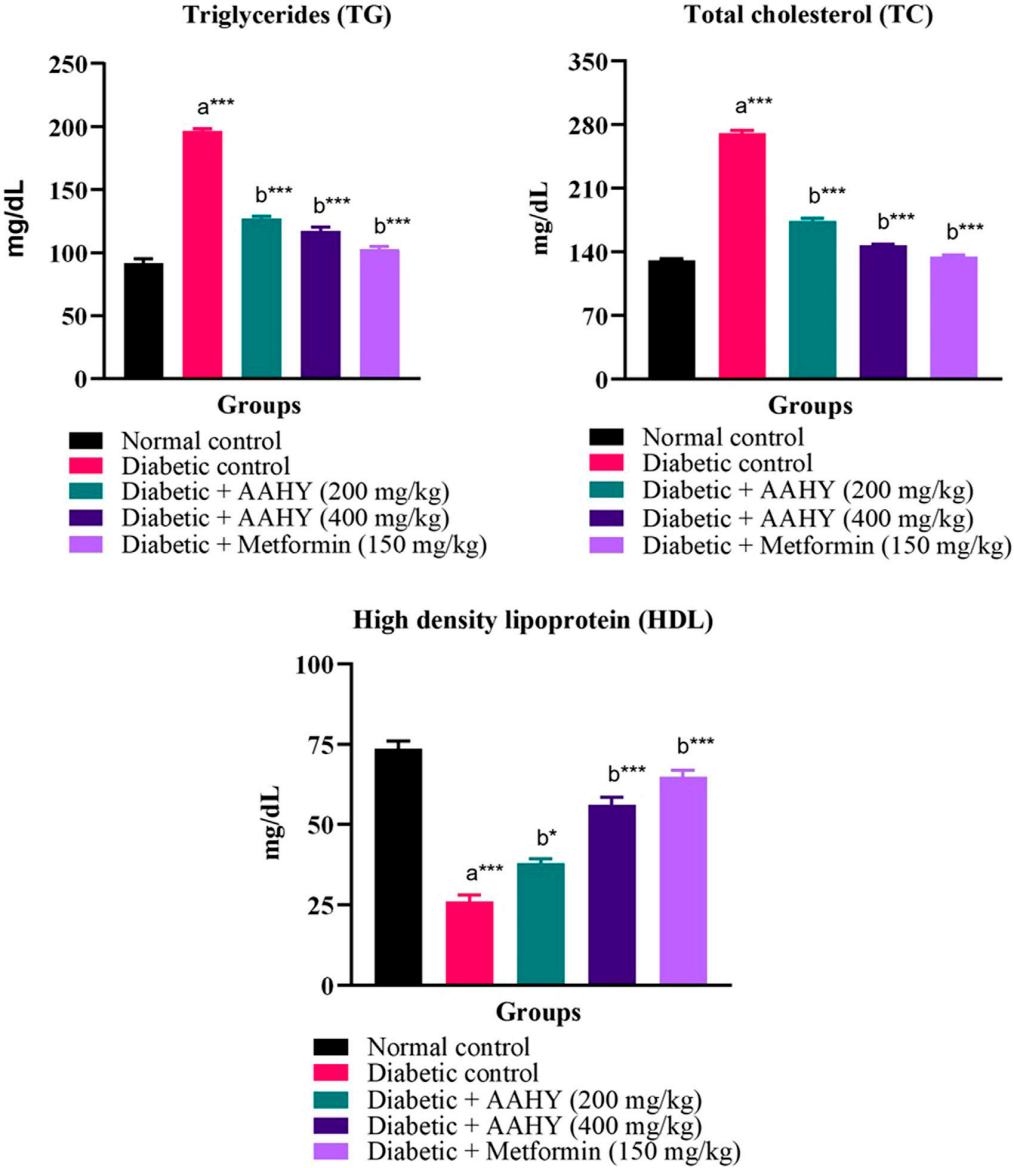
Effect of the AAHY extract on the serum lipid profile. The error bar denotes mean ± SEM and *n* = 6 for each group. a represents the diabetic control group versus the normal control group, and b represents the diabetic control group versus treated groups. *p < 0.05, **p < 0.01, and ***p < 0.001. SEM: standard error of mean.

In STZ–NA-induced diabetic control rats, the serum lipid profile, such as triglyceride and total cholesterol (TC) levels, was high. However, the HDL level was low compared to normal control rats. In diabetic rats, oral administration of the AAHY extract at the doses of 200 and 400 mg/kg b. w. and metformin at 150 mg/ml b. w. showed a gradual reduction in TG and TC levels, but HDL levels increased compared to the diabetic control group (Figure 6).

### 3.13 Tissue antioxidant parameters

**FIGURE 7:**
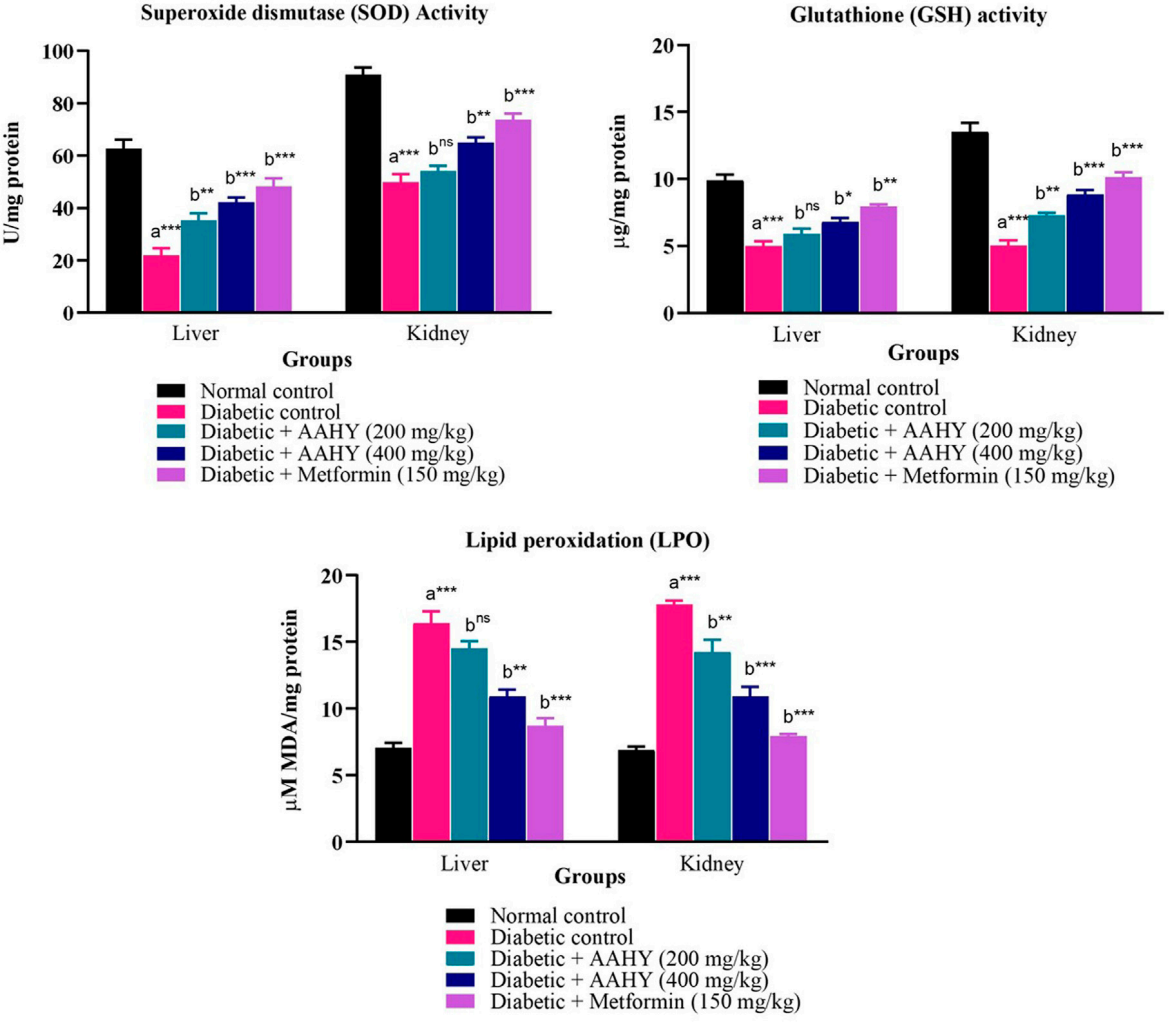
Effect of the AAHY extract on liver and kidney tissue antioxidant parameters. The error bar denotes mean ± SEM, and *n* = 6 for each group. a represents the diabetic control group versus the normal control group, and b represents the diabetic control group versus treated groups. *p < 0.05, **p < 0.01, and ***p < 0.001. SEM: standard error of mean.

The liver and kidney antioxidant data are shown in Figure 7. The SOD and GSH levels of STZ–NA-induced diabetic control rats were low, whereas an increased level of MDA was observed compared to the normal control group. These antioxidant parameters were significantly improved near to normal levels after administering the AAHY extract 200 and 400 mg/kg b.w. doses and metformin at 150 mg/ml b.w, compared to diabetic control rats.

### 3.14 Histopathological studies

**FIGURE 8:**
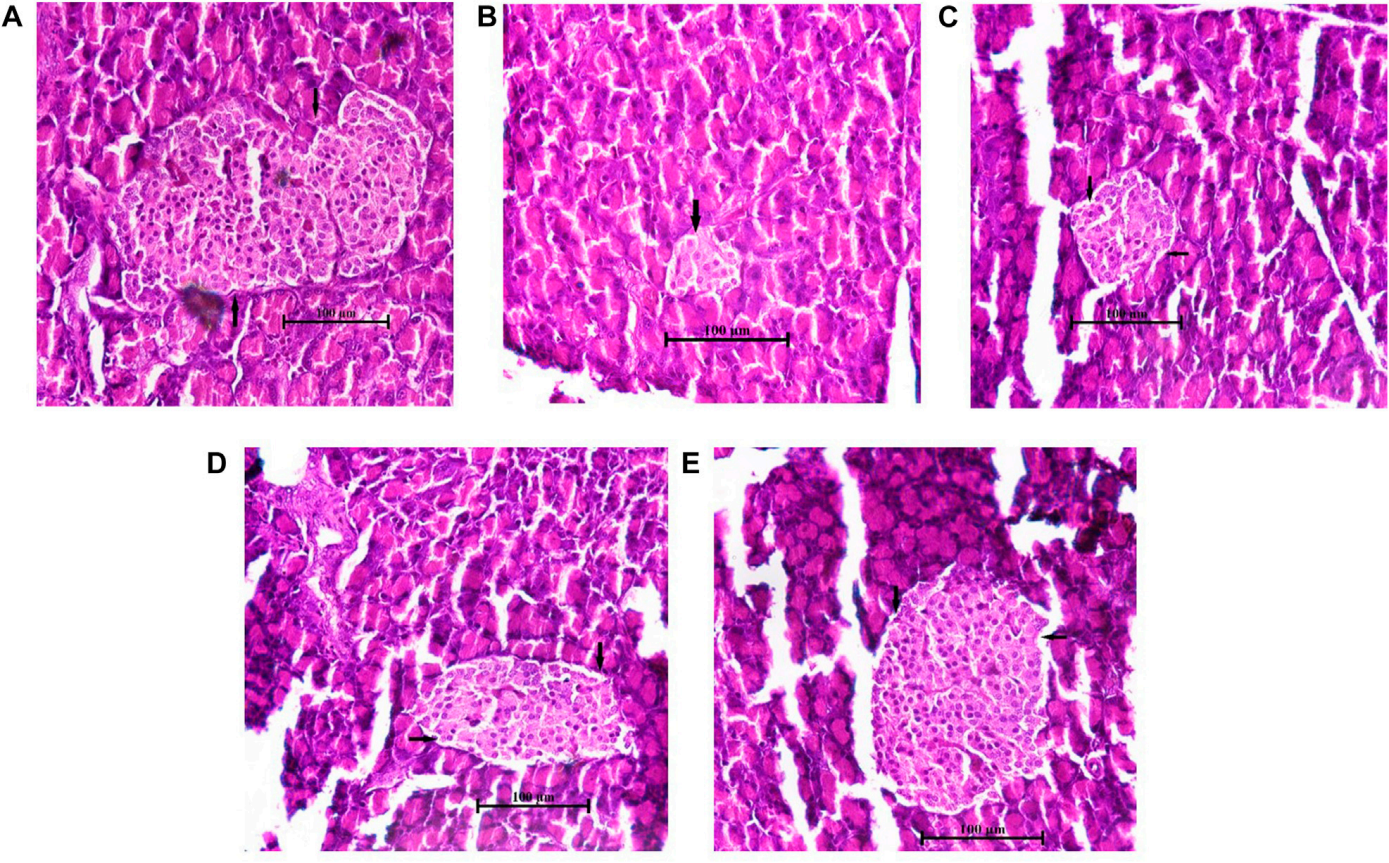
Histopathological features of the pancreatic islets of normal and STZ–NA-induced diabetic Wistar albino rats. **(A)** Normal control group depicting the normal histology of the pancreas, **(B)** diabetic pancreas showing a depleted, distorted β-cell structure and greatly reduced islets size, **(C)** diabetic + AAHY extract 200 mg/kg b.w.-treated pancreas showing mild improvement, **(D)** diabetic + AAHY extract 400 mg/kg b.w.-treated pancreas revealing gradual regeneration of β-cells, thereby improving islets size, and **(E)** diabetic + metformin 150 mg/kg b.w.-treated pancreas showing the nearly normal structure of the islets. A representative photomicrograph of each group is shown (*n* = 6). Scale bar = 100 µm.

**FIGURE 9:**
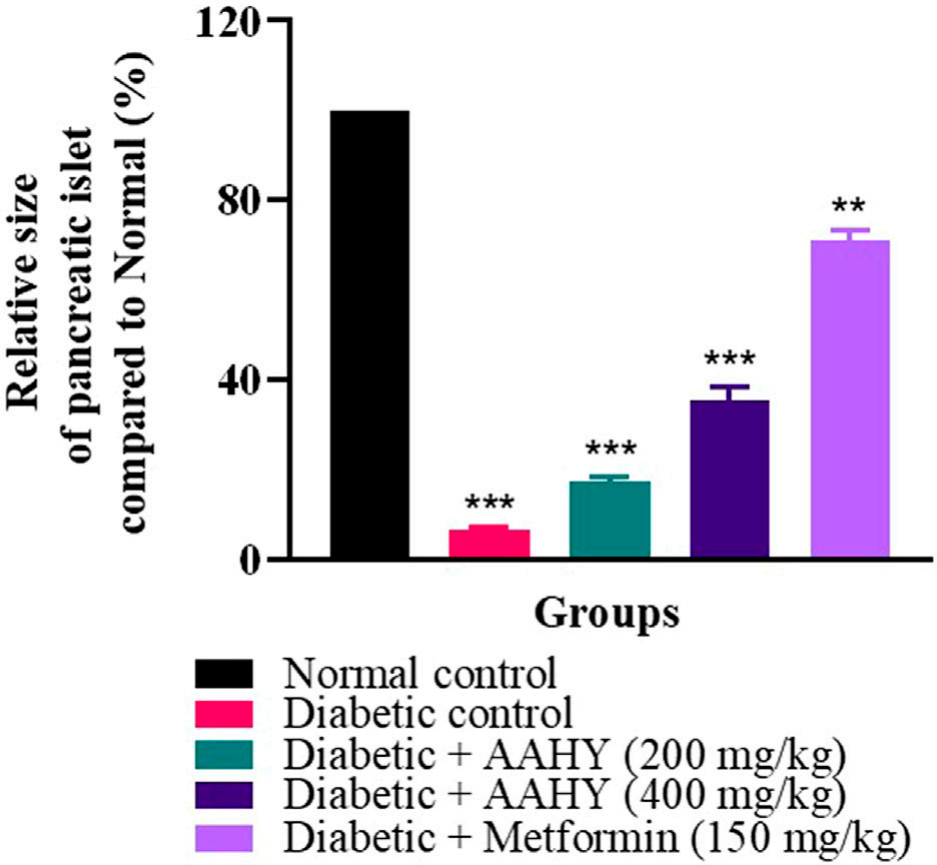
Graph showing the relative size of the pancreatic islets compared to that of the normal control. The error bar denotes mean ± SEM, and *n* = 6 for each group. **p < 0.01 and ***p < 0.001 versus the normal control group. SEM: standard error of mean.

The histology of the pancreas was examined after the end of the experimental design. The hematoxylin and eosin-dyed pancreatic tissue photomicrographs of normal control rats depicted normal islet cells (Figure 8A). On the contrary, the diabetic control pancreatic islet cells were degranulated, disrupted, noticeably depleted, and dilated compared to the normal islet architecture (Figure 8B). However, the AAHY extract 200- and 400 mg/kg b.w.-treated groups exhibited gradual improvement in islet cell density and showed regeneration of cell and granulation in a dose-dependent manner compared to diabetic control rats (Figures 8C, D). The metformin-treated group showed protective response as evident from the increased pancreatic islets size close to the normal histology of the normal control group (Figure 8E). A remarkable difference was observed in the relative pancreatic islets size of the diabetic and treated groups when compared with that of the normal control. The islets sizes in the diabetic group were significantly deduced showing depleted cells (6.78% ± 0.39%). A gradual improvement in the histology of the pancreatic islets was observed in the AAHY extract (17.23% ± 1.25% and 35.46% ± 2.96%, respectively)-treated group and there was a significant improvement in the metformin (71.1% ± 2.04%)-treated group when relatively compared with that of the normal group (Figure 9).

## 4 Discussions

Although there have been significant advances in the development of antidiabetic drugs, these therapies are often regarded as ineffective since they fail to prevent T2DM-related secondary problems, have adverse effects, and are expensive. Therefore, it is encouraging to look into medicinal plants and other natural remedies as therapeutic alternatives.

The current study has been conducted to evaluate the capacity of *Ageratina adenophora* hydroalcoholic extract administered orally, for antihyperglycemic and antihyperlipidemic activities in normal, oral glucose-loaded, and STZ–NA-induced diabetic rats, and quantification of phytochemicals chlorogenic and caffeic acids present in the extract. Before conducting *in vivo* animal studies in Wistar albino rats, *in vitro* enzymatic assays, α-glucosidase and α-amylase, were performed. T2DM is characterized by faulty insulin secretion or deficiency and defective metabolism of carbohydrates and lipids in tissues, leading to an increase in postprandial hyperglycemia levels. The pancreatic enzymes α-glucosidase and α-amylase hydrolyze starch and oligosaccharides resulting in fast uptake of glucose in the intestine, thereby increasing the postprandial blood glucose level. Inhibition of these enzymes is a promising method for reducing such postprandial hyperglycemia, which is crucial in T2DM (Dong et al., 2012). The findings suggest that the AAHY extract possesses good suppressive activities on pancreatic α-glucosidase and α-amylase enzymes, thereby inhibiting carbohydrate metabolism and decreasing the postprandial glucose level.

Prior to the advancement of *in vivo* experiments, the acute toxicity test was performed. Oral administration of the AAHY extract at a dose of 2,000 mg/kg b. w. in mice did not show any toxicity effect. So, further downstream experiments were carried out at doses of 200 and 400 mg/kg b.w, respectively. The oral glucose tolerance test is one of the important assessments for the identification and diagnosis of impaired glucose tolerance, insulin resistance, and sensitivity for T2DM prevention in patients who are at high risk (Kuo et al., 2021). So, it can be interpreted that the AAHY extract has a significant glucose tolerance activity compared to the normal control groups. Oral administration of the AAHY extract at doses of 200 and 400 mg/kg b.w. for consecutive 28 days significantly reduced FBG compared to the diabetic control groups. This might be due to the potential of the extract to induce pancreatic secretion of insulin by the existing and regenerated β-cells. The body weight of the STZ–NA-induced diabetic rats was decreased compared to normal group rats, which could be possibly due to low glycemic grade. In untreated diabetic rats, there is an extensive breakdown of proteins to provide amino acids for gluconeogenesis during insulin deficiency resulting in muscle wasting and weight loss (Kasetti et al., 2010). The body weight of the treated diabetic groups gradually improved after the administration of the AAHY extract.

Antioxidants play a protective role against the development of many chronic diseases caused by the overproduction of oxidants. Antioxidant phytochemicals are present in several medicinal plants that can scavenge free radicals (Yao et al., 2013). Plant polyphenolic compounds are known to possess antioxidant properties and modulate the hyperglycemic state by inhibiting α-amylase and α-glucosidase activities, thereby managing T2DM (Sekhon-Loodu and Rupasinghe, 2019). Chlorogenic and caffeic acids are natural antioxidant phenolic compounds that prevent oxidative stress-induced diseases such as DM (Stagos, 2020). Chen *et al.* reported the synergistic effect of chlorogenic and caffeic acids isolated from *Sonchus oleraceus* Linn. in modulating glucose utilization via the PI3K/AKT/GLUT4 inactivation pathway in HepG2 cells (Chen et al., 2019). The phenolic compound, chlorogenic acid, is found to be abundant in the leaves of *A. adenophora* (Liu et al., 2016). Quantification of chlorogenic acid and its associate, caffeic acid, was standardized using HPTLC analysis and was found to be 6.47% w/w and 3.28% w/w, respectively. The antioxidant and antiinflammatory properties of these compounds are well reported and also aid in the management of chronic metabolic diseases (Dos Santos et al., 2006; Santana-Gálvez et al., 2017). Previous research showed a variety of plant polyphenolics possessing antioxidant properties that protect pancreatic β-cells (Belayneh et al., 2019). Similarly, the AAHY extract showed antioxidant property in the present study, suggesting the ability to protect β-cells and contribute to antihyperglycemic activity in STZ–NA-induced diabetic rats. The protective role might be attributed to the presence of various groups of secondary metabolites such as alkaloids, phenolics, flavonoids, tannins, glycosides, terpenoids, and quinones, as revealed from the preliminary qualitative profiling of phytochemicals of the AAHY leaf extract (Zhang et al., 2015). The aforementioned phytoconstituent results are also consistent with the reported studies (André et al., 2019; Poudel et al., 2020). The alkaloids have been reported to regulate hyperglycemia via α-glucosidase and α-amylase inhibition, sensitizing insulin production, inhibition of PTP-1B and DPP-4 pathways, and managing oxidative stress condition (Adhikari, 2021; Ajebli et al., 2021). Terpenoids also help in the management of T2DM via the activation of the AMP-activated protein kinase pathway (Grace et al., 2019). Phenolics and flavonoids are polyphenols possessing antioxidant properties that protect the pancreatic islets of Langerhans (Praparatana et al., 2022). Likewise, tannins (Ajebli and Eddouks, 2019), glycosides (Sumaira and Khan, 2018), and quinones (Demir et al., 2019) have also been reported to have a hypoglycemic effect. These phytochemicals offer significant potential for metabolic homeostasis. Thus, the presence of these secondary metabolites might have synergistically contributed to the antihyperglycemic effect of the AAHY extract. Despite several *in vitro* and *in vivo* experiments demonstrating the promising therapeutic role of bioactive compounds, only few have reached clinical trials (Adhikari, 2021). So, based on the findings, proper evaluation of the diverse functions of these phytochemicals is still critically required for advancement in antidiabetic drug discovery.

The key factors for the pathogenesis and advancement of DM are associated with oxidative stress and inflammation of the pancreas. Induction of diabetes by STZ–NA in rats caused grave damage to pancreatic β-cells due to excessive generation of free radicals such as reactive oxygen and nitrogen species (Bhatti et al., 2022). In consistency with the aforementioned study, the key antioxidant enzymes’, SOD and GSH, levels were significantly reduced, while lipid peroxidation levels were elevated as evidenced by the increased MDA, in the liver and kidney tissues of the diabetic control group, which is an indication of STZ–NA-induced oxidative stress. However, treatment of the AAHY extract 200 and 400 mg/kg b.w. doses and metformin at 150 mg/ml b.w significantly improved the enzyme levels comparable to normal levels, demonstrating the capabilities of the AAHY extract and metformin to decrease oxidative stress.

Glycated hemoglobin (HbA1c) concentration in the blood is a reliable diagnostic marker for the determination of diabetes (WHO, 2011). The American Diabetes Association (ADA) suggested HbA1c level ≥6.5% as a high-risk glycemic state and is directly proportional to the blood plasma glucose content. HbA1c indicates a cumulative history of blood glucose levels over the last 2–3 months (Sherwani et al., 2016). However, the threshold point of the HbA1c test is still controversial among different expertised organizations. HbA1c is influenced by several physiological and pathological conditions. So, the test is preferably taken in conjunction with other tests such as the fasting blood glucose and oral glucose tolerance test for appropriate diagnosis (Hussain, 2016). In the present experimental study, the OGTT and FBG test showed reduction in blood glucose levels after administration of the AAHY extract and metformin. HbA1c was also reduced in the treated groups compared to that of the diabetic group. The decrease in HbA1c and FBG levels in treated diabetic rats is an indication of improving glycemia.

The liver is the largest vital organ for metabolism, excretion, and detoxification. Liver damage is linked with necrotic cells, a hike in tissue lipid peroxidation, and a decrease in reduced glutathione levels, in addition to an increase in serum biochemical markers such as SGPT, SGOT SALP, triglycerides, and cholesterol (Abou Seif, 2016). The liver of the STZ–NA-induced rats was damaged, and thus, the elevated serum biochemical markers of liver function might be primarily due to the enzymes leaking from the liver cytosol into the bloodstream (Kasetti et al., 2010). Administration of the extract and metformin showed significant reduction in the serum biochemical parameters compared to the diabetic control groups. The diabetic control rats showed the presence of significantly elevated levels of urea and creatinine in serum, which are known markers for kidney dysfunction (Gowda et al., 2010). The serum urea and creatinine levels, presented in the current study of the diabetic treated rats were reduced, indicating the gradual improvement of renal damage compared to the untreated rats.

Abnormal metabolism of enzyme lipoprotein lipase leads to accumulation of triglycerides and total cholesterol (TC), but decreased HDL cholesterol, which are commonly linked with DM and result in diabetic dyslipidemia. Increased levels of TG and TC and decreased HDL are also significant risk factors for cardiovascular diseases (CVDs). Elevated HDL aids in transportation of cholesterol to the liver, which is the primary site for fatty acid metabolism, thus reducing the risk of CVDs. So, in the diabetic condition, there is insulin deficiency due to which lipoproteins are unable to hydrolyze the lipids resulting in the systemic imbalance of synthesis, release, and rate of clearance of lipids (Moodley et al., 2015) (Sisay et al., 2022). In consistency with the reported data, we found elevated levels of TG and TC and decreased HDL cholesterol in the untreated STZ–NA-induced diabetic control group. Administering daily doses of the AAHY extract and metformin significantly decreased TG and TC, while increasing HDL cholesterol in the diabetic rats, indicating that the plant extract improves diabetes dyslipidemic conditions.

Histopathological examination of the STZ–NA-induced diabetic pancreas showed drastic damage to the β-cells, thereby reducing the number and size of the islets. The AAHY extract and metformin treatment groups improved the structure, number, and size of the pancreatic islets. The relative sizes of the treated pancreatic islets increased from 6.78% ± 0.39% in diabetic to 17.23% ± 1.25% and 35.46% ± 2.96% in the AAHY extract 200 and 400 mg/kg b.w.-treated groups when compared with the normal group. Regeneration and restoration of normal β-cell function are critically required for a successful prevention and treatment of diabetes (Chen et al., 2017). A gradual improvement observed in the pancreatic histology of treatment groups suggests the potential preventive and protective nature of the extract against STZ-induced diabetic rats. However, the mechanistic pathway whether the effective nature was due to insulin production or sensitization following administration of the AAHY extract needs to be further assessed.

## 5 Conclusion

From this study, it can be concluded that the hydroalcoholic extract of *A. adenophora* has significant blood glucose lowering capacity on nomoglycemic, diabetic, and oral glucose-loaded Wistar albino rats while maintaining the body weight of diabetic rats. The experimental results verified that the *A. adenophora* hydroalcoholic extract has beneficial effects in inhibiting α-glucosidase and α-amylase activities and restored the altered blood glucose level, glycated hemoglobin, body weight, serum enzymes (SGOT, SGPT, and ALP), total protein, urea, and creatinine levels close to the normal range in treated STZ–NA-induced diabetic rats. The AAHY extract significantly enhanced tissue antioxidant parameters (SOD, GSH, and LPO) close to the normal level. The presence of high quantity of chlorogenic and caffeic acids as some of the major phytoconstituents may contribute to the improvement of glucose tolerance, insulin resistance, cellular oxidative stress, and obesity in STZ-induced diabetic rats. However, purification of chlorogenic and caffeic acids, as well as their novel mechanisms of lowering the hyperglycemic condition in T2DM, needs detailed evaluation. In addition, the bioactivity of other groups of secondary metabolites alone or synergistic effect needs scientific evaluation. Even though the results provide scientific support for the traditional use of the plant to treat diabetes, elaborative research is obligatory for safety assessment in the long run. Although the preventive role of the AAHY extract against T2DM is undeniable, further pharmacokinetic and mechanistic pathway studies are needed to determine the extract metabolism, normalization of blood glucose, and biochemical parameters, as well as insulin production or sensitization following administration of the AAHY extract. The present study could play a promoting role in the discovery and development of a new antidiabetic agent from *A. adenophora*.

## Data Availability

The original contributions presented in the study are included in the article/Supplementary Material; further inquiries can be directed to the corresponding author.
